# Screening with Magnetic Resonance Imaging in women at low and intermediate risk of breast Cancer

**DOI:** 10.1186/1897-4287-10-S4-A18

**Published:** 2012-12-10

**Authors:** T T Huzarski, B Górecka-Szyld, J J Huzarska, G Psut, G Wilk, R Sibilski, C Cybulski, B Kozak-Klonowska, M Siołek, E Kilar, D Czudowska, H Janiszewska, D Godlewski, A Mackiewicz, J Jarkiewicz-Tretyn, I Szabo-Moskal, J Gronwald, J Lubiński, SA Narod

**Affiliations:** 1 Department of Genetics and Pathology, International Hereditary Cancer Center, Pomeranian Medical University, Szczecin, Poland; 2 Department of Radiology, Pomeranian Medical University, Szczecin, Poland; 3 Euro-Medic Diagnostics Poland Ltd, Szczecin, Poland; 4 Oncology Diagnostic Center, Zielona Góra, Poland; 5 Regional Oncology Center, Kielce, Poland; 6 Department of Oncology, District Specialist Hospital, Świdnica, Poland; 7 Oncology Diagnostic Center, Legnica, Poland; 8 Department of Clinical Genetics, Collegium Medicum, Nicolaus Copernicus University, Bydgoszcz, Poland; 9 Prophylactic and Epidemiology Cancer Center, Poznań, Poland; 10 Department of Cancer Immunology, Medical University, Poznań, Poland; 11 District Specialist Hospital, Toruń, Poland; 12 Department of Radiology, Regional Oncology Hospital, Bydgoszcz, Poland; 13 Women’s College Research Institute, Women’s College Hospital and the University of Toronto, Canada

## Purpose

The addition of MRI to mammography and ultrasound for breast cancer screening has been shown to improve screening sensitivity in high risk women (i.e., those with a BRCA mutation). Here we evaluate the addition of MRI to conventional screening (ultrasound and mammography) for women at average or intermediate risk of cancer.

## Patients and Methods

From 2008 to 2011, 2995 women, aged 40 to 65 years with no previous history of breast cancer were enrolled in a prospective screening trial consisting of two annual rounds of MRI, ultrasound and mammography. 356 women had a CHEK2 mutation, 458 women had a first-degree relative with breast cancer and 2269 women had neither risk factor. Subjects were followed for incident cancer for one year from the date of the second screening examination.

## Results

In this cohort of 2995 women, 21 invasive epithelial cancers, one angiosarcoma and four cases of DCIS were identified over a two-year period. Of the invasive cancers, 20 were screen-detected and one was an interval cancer. Of the 21 invasive cancers detected in the cohort, 14 (67%) were less than 2 cm and 16 (76%) were node-negative. The sensitivity of MRI was 90%, the sensitivity of ultrasound was 62% and the sensitivity of mammography was 57%. The number of biopsies incurred by MRI (156) was far greater than the number incurred by either mammography (n = 35) or by ultrasound (n = 57). No cancer was identified by mammography that was not also identified by MRI, but one cancer was detected by ultrasound that was missed by MRI. Of the 19 cancers that were detected by MRI, 17 were also detected by ultrasound or mammography and two were detected by MRI alone.

## Conclusion

In terms of sensitivity, MRI is superior or similar to the combination of mammography and ultrasound for screening of women at low or intermediate risk of breast cancer. However, because of the additional costs incurred and the number of biopsies required in order to detect a few additional breast cancers, MRI screening is probably not warranted outside of high-risk populations.

